# Sim1 Is a Novel Regulator in the Differentiation of Mouse Dorsal Raphe Serotonergic Neurons

**DOI:** 10.1371/journal.pone.0019239

**Published:** 2011-04-26

**Authors:** Nadja Osterberg, Michael Wiehle, Oliver Oehlke, Stefanie Heidrich, Cheng Xu, Chen-Ming Fan, Kerstin Krieglstein, Eleni Roussa

**Affiliations:** 1 Department for Neuroanatomy, Georg-August-University Goettingen, Goettingen, Germany; 2 DFG Research Center Molecular Physiology of the Brain (CMPB), Goettingen, Germany; 3 Anatomy and Cell Biology, Department of Molecular Embryology, Albert-Ludwigs-University Freiburg, Freiburg, Germany; 4 Department of Embryology, Carnegie Institution, Baltimore, Maryland, United States of America; Tokyo Medical and Dental University, Japan

## Abstract

**Background:**

Mesencephalic dopaminergic neurons (mDA) and serotonergic (5-HT) neurons are clinically important ventral neuronal populations. Degeneration of mDA is associated with Parkinson's disease; defects in the serotonergic system are related to depression, obsessive-compulsive disorder, and schizophrenia. Although these neuronal subpopulations reveal positional and developmental relationships, the developmental cascades that govern specification and differentiation of mDA or 5-HT neurons reveal missing determinants and are not yet understood.

**Methodology:**

We investigated the impact of the transcription factor *Sim1* in the differentiation of mDA and rostral 5-HT neurons *in vivo* using *Sim1-/-* mouse embryos and newborn pups, and *in vitro* by gain- and loss-of-function approaches.

**Principal Findings:**

We show a selective significant reduction in the number of dorsal raphe nucleus (DRN) 5-HT neurons in *Sim1-/-* newborn mice. In contrast, 5-HT neurons of other raphe nuclei as well as dopaminergic neurons were not affected. Analysis of the underlying molecular mechanism revealed that tryptophan hydroxylase 2 (*Tph2*) and the transcription factor *Pet1* are regulated by *Sim1.* Moreover, the transcription factor *Lhx8* and the modulator of 5-HT_1A_-mediated neurotransmitter release, *Rgs4,* exhibit significant higher expression in ventral hindbrain, compared to midbrain and are target genes of *Sim1*.

**Conclusions:**

The results demonstrate for the first time a selective transcription factor dependence of the 5-HT cell groups, and introduce Sim1 as a regulator of DRN specification acting upstream of *Pet1* and *Tph2*. Moreover, Sim1 may act to modulate serotonin release via regulating RGS4. Our study underscores that subpopulations of a common neurotransmitter phenotype use distinct combinations of transcription factors to control the expression of shared properties.

## Introduction

Cell and neuron subpopulations in the CNS differentiate in stereotypic, defined positions along the anterior-posterior and dorso-ventral axes of the neural tube and exhibit a cell-type-specific transcriptional code [1]. Among neuronal subpopulations, mesencephalic dopaminergic neurons (mDA) and serotonergic (5-HT) neurons are of particular interest, because of their involvement in neurological and degenerative diseases. Degeneration of mDA is associated with Parkinson's disease [2], defects in the serotonergic system are related with depression, obsessive-compulsive disorder, and schizophrenia [3].

During development of the CNS, mDA and 5-HT neurons are among the first to emerge [4]. Because of their early onset 5-HT neurons are even regarded to play a role in several differentiation processes, acting as a growth and differentiation promoting factor in addition to being a neurotransmitter. 5-HT neurons are involved in the differentiation of precursor cells in their target regions. Both mDA and 5-HT neurons develop as distinct cell groups and have a close ontogenetic relationship: their respective *Otx2*- and *Gbx2*-expressing progenitors are located adjacent on either side of the midbrain-hindbrain boundary [5], their development critically depends on Shh and FGF8 [6], and the transcriptional code controlling differentiation of progenitors toward dopaminergic or serotonergic cell fate shares common determinants, such as *Nkx2.2, Lmx1b,* and *Mash1* [7]–[9]. However, the transcriptional equipment of mDA additionally includes *Lmx1a, Nurr1, Pitx3*, and *En1/2* [10], whereas differentiation of rostral 5-HT neurons involves *Pet1* and *Gata2* [11], [12]. In spite of numerous studies, the transcriptional network and the developmental cascades that govern specification and early differentiation of dopaminergic or serotonergic neurons reveal missing determinants, and are not yet understood.

The *Drosophila* CNS midline cells derive from the mesectoderm that gives rise to CNS midline progenitor cells, and their development requires expression of the bHLH/PAS domain transcription factor *single-minded* (sim) [13]–[16]. Thereby, *sim* activates expression of *rho*, *en*, and *egfr* [17]. The two mammalian homologs, *Sim1* and *Sim2* [18], reveal differential expression patterns during mouse embryogenesis [19]. *Sim1-/-* and *Sim2-/-* mice die perinatally due to hypothalamic and respiratory defects, respectively [20], [21]. Interestingly, *Gata2*, expressed in postmitotic serotonergic progenitors, and *Gdnf*, a survival promoting factor for mDA [22], represent target genes of *Sim1* [23]. Moreover, Sim1 has shown to be upregulated in mouse ventral regions of the CNS (M. Wiehle, N. Osterberg, and E. Roussa, unpublished observations).

Here we demonstrate the impact of *Sim1* in the differentiation of mDA and rostral 5-HT neurons using *Sim1* gain- and loss-of-function experiments *in vitro*, and *Sim1* deficient mice *in vivo*. The results show that development of mDA is Sim1-independent. Conversely, Sim1 represents an important regulator in the development of a subpopulation of rostral 5-HT neurons, the dorsal raphe nucleus, acting upstream of *Pet1* and *Tph2*. Moreover, we have identified target genes of *Sim1*, namely *Lhx8, RGS4,* and *Brn3.2* being upregulated in ventral hindbrain, compared to ventral midbrain, and *Lhx8* and *RGS4* as putative candidates involved in the specification of mouse rostral serotonergic neurons.

## Results

### *Sim1* is expressed in mouse ventral midbrain and ventral hindbrain and co-localizes with TH and 5-HT

**Figure 1:**
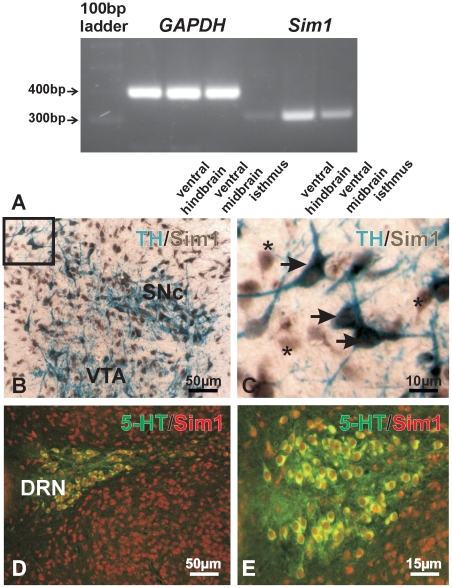
Sim1 mRNA expression by RT-PCR and cellular localization of Sim1 in mouse tissues by immunohistochemistry. A: *Sim1* transcript was detected in mouse E12 ventral hindbrain, ventral midbrain, and isthmus region using *sim1* primers corresponding to nucleotides (nt) 736-756 and nt:1052-1032 of the mouse Sim1 gene (Genbank accession number: NM011376). B: double immunolabeling on mouse fixed paraffin sections for TH (blue) and Sim1 (brown) showed Sim1 localization in the cell nucleus and cytoplasmic staining pattern for TH in midbrain substantia nigra pars compacta (SNc) and ventral tegmental area (VTA). Inset at higher magnification shown in C: co-localization of TH and Sim1 is apparent. Arrows point to cells that are immunopositive for both TH and Sim1. Asterisks indicate Sim1 positive, but TH negative cells. D, E: double immunofluorescence for 5-HT (green) and Sim1 (red) in mouse hindbrain revealed co-localization of the proteins.

As the first step to investigate potential involvement of Sim1 in the differentiation of mDA and 5-HT neurons, we have determined that *Sim1* is expressed in these brain areas at crucial developmental stages. Fig. 1A shows RT-PCR analysis of mouse E12 ventral midbrain, isthmus, and ventral hindbrain tissue using specific Sim1 primers. A transcript of the expected size of ∼319 bp could be amplified from all probes examined (Fig. 1A, lanes 5–7). *Sim1* expression was considerably stronger in mouse ventral midbrain, compared to hindbrain. Subsequently, cellular localization of Sim1 with the late markers for dopaminergic and serotonergic lineage, TH and 5-HT, respectively, has been performed in newborn mice. Fig. 1B illustrates double immunolabeling for TH and Sim1 on fixed paraffin sections from mouse midbrain at the area of substantia nigra pars compacta (SNc) and ventral tegmental area (VTA). Sim1 was exclusively expressed in cell nuclei, whereas TH showed an intracellular labeling pattern. At higher magnification, a co-localization of Sim1 could be observed with TH (Fig. 1C). However, whereas all TH positive neurons showed additionally immunoreactivity for Sim1 (Fig. 1C, arrows), many Sim1 immunopositive cells were devoid of TH immunoreactivity (Fig. 1C, asterisks), indicating a broader expression domain of Sim1. Similar results could be obtained in all rostral serotonergic neurons of newborn mice. Fig. 1D and 1E exemplarily illustrate double immunofluorescence for Sim1 (red) and 5-HT (green) in the area of dorsal raphe nucleus (DRN). Sim1 was found always co-localized with 5-HT neurons. Again, in several Sim1 positive cells 5-HT immunoreactivity could not be detected.

### *Sim1* is required for the differentiation of a subpopulation of mouse rostral serotonergic neurons, but not for mesencephalic dopaminergic neurons

In *Drosophila melanogaster,* Sim is required for development of all midline CNS cells [24]. We have investigated putative effects of the mouse homolog *Sim1* in the induction and specification of ventral neuronal subpopulations, represented by mDA and rostral 5-HT neurons, *in vivo.*

**Figure 2:**
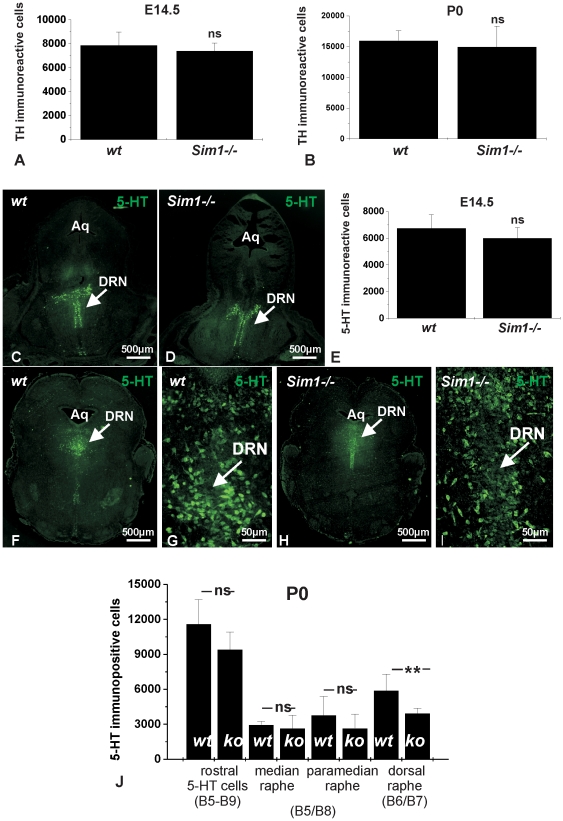
Phenotype analysis for midbrain dopaminergic and rostral serotonergic neurons of *Sim1-/-* mutants. Counting of TH positive cells after immunostaining in midbrain tissue sections revealed no differences in the number of midbrain dopaminergic neurons between wild type (*wt*) and *Sim1-/-* mutants at embryonic day (E)14.5 (A) and newborn (P0; B). ns: not significant. C-D: 5-HT immunofluorescence in frontal sections of *wt* (C) and *Sim1-/-* embryos (D) at E14.5 (DRN: dorsal raphe nucleus; Aq: aqueduct). E: Quantification of cell counts: numbers of 5-HT immunopositive cells were comparable between *wt* and *Sim1-/-*. F-J: Whereas quantification of cell counts revealed no statistical differences (ns) in the total number of rostral 5-HT neurons, median raphe, and paramedian raphe 5-HT neurons between *Sim1-/-* (H) and their wt littermates (F), the number of 5-HT immunoreactive cells in the dorsal raphe nucleus (DRN) was found significantly decreased in *Sim1-/-*, compared to the wild type. 5-HT immunolabeling in mouse rostral raphe nuclei at P0 in *wt* (G) and *Sim1* mutants (I). Arrow points to dorsal raphe nucleus (DRN). Aq: aqueduct.

Fig. 2A shows cell counts after immunohistochemistry of midbrain TH immunopositive cells of fixed wild type (*wt*) and *Sim1-/-* mutant mice embryos at E14.5. The results show no significant differences in the number of midbrain TH immunoreactive neurons between *wt* and *Sim1-/-* mutant embryos (*p* = 0.4023, n = 6). Similar results could be obtained in newborn mice (P0) for TH immunoreactive cells of the substantia nigra and ventral tegmental area (Fig. 2B; *p* = 0.5220, n = 6).

Analysis of the serotonergic system from *wt* (C) and *Sim1-/-* mutants (D) at E14.5 showed that the total number of 5-HT immunolabeled rostral serotonergic neurons was comparable between *Sim1-/-* embryos and their *wt* littermates (Fig. 2E; *p* = 0.15, n = 6). Similarly, in newborn mice (Fig. 2J), the total number of rostral 5-HT immunopositive cells (i.e. raphe nuclei B5-B9) showed no significant differences between *wt* (Fig. 2F) and *Sim1-/-* (Fig. 2H; *p* = 0.06, n = 6). However, when 5-HT immunoreactive neurons were quantified for each nucleus separately, a significant decrease in the number of serotonergic neurons in the dorsal raphe nucleus (DRN) was found in *Sim1-/-* (Figs. 2I), compared to the *wt* (Figs. 2G; **p = 0.009, n = 6). In contrast, the number of 5-HT immunopositive cells in the median and paramedian raphe nuclei were comparable between *wt* and *Sim1* mutants (Fig. 2J; *p* = 0.61 and *p* = 0.27, respectively, n = 6).

These data implicate a selective role for Sim1 in the differentiation of a particular subset of rostral 5-HT neurons, whereas induction, specification, and maintenance of mDA are Sim1-independent.

### MN9D cell line expresses dopaminergic and serotonergic markers

Elucidation for the role of a gene of interest in the development of mDA and 5-HT neurons can be complemented by an adequate *in vitro* system that allows gain of function and loss of function experiments. The MN9D cell line [25] expresses key dopaminergic markers and has been used in studies dealing with differentiation of dopaminergic neurons [26].

**Figure 3:**
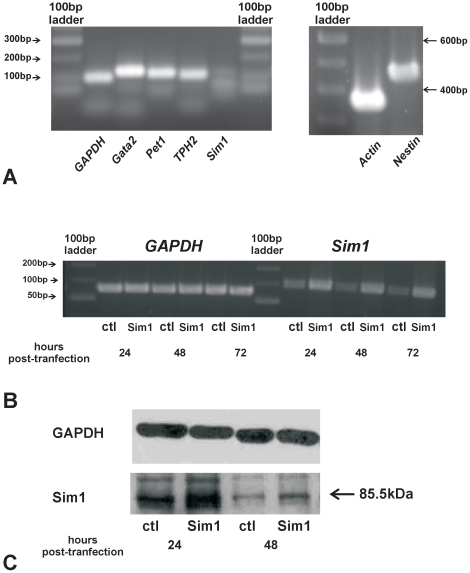
MN9D cells, a suitable *in vitro* model system. A: Expression of determinants of the serotonergic lineage in the MN9D cell line by RT-PCR. *Gata2*, *Pet1*, and *Tph2* could be amplified from MN9D cells cDNA. The Sim1 primers used correspond to nucleotides (nt) 37-66 and nt:152-126 of the mouse *Sim1*. In addition, expression of the neural stem cell marker *nestin* was also detected. B: RT-PCR analysis of *Sim1* expression in MN9D cells cDNA, 24 hours, 48 hours, and 72 hours after transfection of the cells with either pcDNA3::Sim1 or pcDNA3 expression vectors (ctl) and GAPDH as housekeeping gene. *Sim1* expression was clearly increased in pcDNA3::Sim1-transfected MN9D cells, compared to the pcDNA3-transfected cells. C: Sim1 protein abundance by immunoblotting in homogenates of MN9D cells transfected either with pcDNA3::Sim1 or pcDNA3 expression vectors (ctl) 24 hours and 48 hours after transfection. The immunoblots were probed either with monoclonal antibody against GAPDH or with rabbit polyclonal antibody against Sim1. Sim1 protein was significantly upregulated in pcDNA3::Sim1-transfected MN9D cells, compared to the pcDNA3-transfected cells (**P<0.01 after densitometric analysis of the signal ratio Sim1: GAPDH and Student's *t*-test, n = 3). Arrow points to the band of the expected size. The blots are representative for three different experiments. 30 µg protein was loaded per lane.

However, MN9D cells apparently express specific markers for the serotonergic lineage as well, as assessed by RT-PCR analysis, shown in Fig. 3A. Transcripts of the expected size for *Gata2* (∼110 bp, lane 3), expressed in mitotic and postmitotic serotonergic progenitors, and for *Pet1* (∼109 bp, lane 4), exclusively expressed in postmitotic 5-HT neurons, could be amplified from MN9D cells cDNA. In addition, expression of *TPH2* (∼99 bp, lane 5), a late specific marker for the serotonergic lineage, was also found. The gene of interest, *Sim1*, showed only weak expression level in MN9D cell line (∼115 bp, lane 6). Moreover, besides genes involved in induction of 5-HT phenotype, expression of *Nestin* (∼438 bp, lane 10), a marker for neural precursor cells, was also expressed, indicating that the MN9D cell line is comprised of a mixed cell population. Taken together, these results indicate that MN9D cells represent a suitable *in vitro* model system to study the molecular mechanisms underlying differentiation of mDA and 5-HT neurons.

Fig. 3B illustrates RT-PCR analysis after overexpression of *Sim1* in MN9D cells 24 h, 48 h, and 72 h after transfection of the cells either with pcDNA3 expression vector (controls; ctl) or with pcDNA3::Sim1 expression vector (Sim1), as described in Material and Methods. *Sim1* was expressed at high levels 24 h after transfection with pcDNA3::Sim1, compared to the controls, and remained at this level up to 72 h after transfection. Consistently, western blot analysis using a specific anti-Sim1 antibody (Fig. 3C) showed a prominent immunoreactive band at ∼85.5 kDa (arrow), corresponding to the molecular mass of full length Sim1. As shown in Fig. 3C, in pcDNA3::Sim1-transfected MN9D cells Sim1 protein is abundantly expressed 24 h (lane 2), and 48 h (lane 4) after transfection, compared to the MN9D cells that had been transfected with pcDNA3 (lanes 1 and 3) expression vector. Similar results were obtained 72 h after transfection (data not shown).

### Expression of early and late dopaminergic markers is not affected by *Sim1*

We next examined whether Sim1 over-expression in the MN9D cells has an impact on the expression of genes associated with mDA or rostral 5-HT neurons.

**Figure 4:**
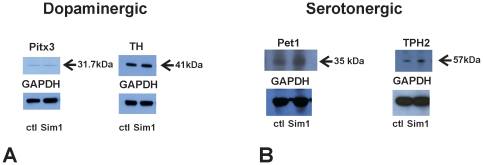
Impact of Sim1 on protein abundance of early and late markers of the dopaminergic (A) and serotonergic (B) lineage. Immunoblotting in homogenates of MN9D cells transfected either with pcDNA3::Sim1 or pcDNA3 expression vectors (ctl) 24 hours after transfection. The immunoblots were probed either with an antibody against GAPDH or with Pitx3, TH, Pet1, or TPH2 antibody. Pitx3 and TH protein expression was comparable between controls and pcDNA3::Sim1-transfected MN9D cells. In contrast, Pet1 and TPH2 protein abundance was significantly upregulated in pcDNA3::Sim1-transfected MN9D cells, compared to the pcDNA3-transfected cells. (*P<0.05 after densitometric analysis of the signal ratio Pet1:GAPDH or TPH2:GAPDH and Student's *t*-test; n = 3). The blots are representative for three different experiments. 30 µg protein was loaded per lane.

Fig. 4A represents the western blot analysis of the MN9D homogenate from the controls, i.e. cells transfected with pcDNA3 expression vector (ctl) and from Sim1-overexpressing cells 24 h after transfection, using anti-GAPDH as a housekeeping gene and Pitx3 or TH antibodies. Protein abundance of the dopaminergic markers Pitx3 (∼31.7 kDa, lanes 1 and 2) and TH (∼41 kDa, lanes 3 and 4) was comparable between controls and Sim1-overexpressing cells (1.16±0.50 fold; *p* = 0.89, n = 3 and 1.27±0.35 fold *p* = 0.72, n = 5, for Pitx3 and TH, respectively). Similar results were obtained in probes collected 48 h and 72 h after transfection (data not shown).

Fig. 4B illustrates a representative immunoblot of homogenate from MN9D controls and MN9D overexpressing Sim1, probed with specific anti-Pet1 and anti-TPH2 antibodies. Using the anti-Pet1 and anti-TPH2 antibody respective bands at ∼35 kDa and at ∼57 kDa could be detected in both samples, corresponding to the respective full length proteins. However, both Pet1 and TPH2 protein was significantly upregulated in MN9D cells overexpressing Sim1, compared to the MN9D cells that had been transfected with pcDNA3 vector (2.15±0.32 fold, *p = 0.04 and 2.68±0.41 fold, *p = 0.031, n = 4 for Pet1 and TPH2, respectively).

### Expression of serotonergic markers *Pet1* and *Tph2*, but not *Mash1* and *Gata2*, are controlled by *Sim1*

**Figure 5:**
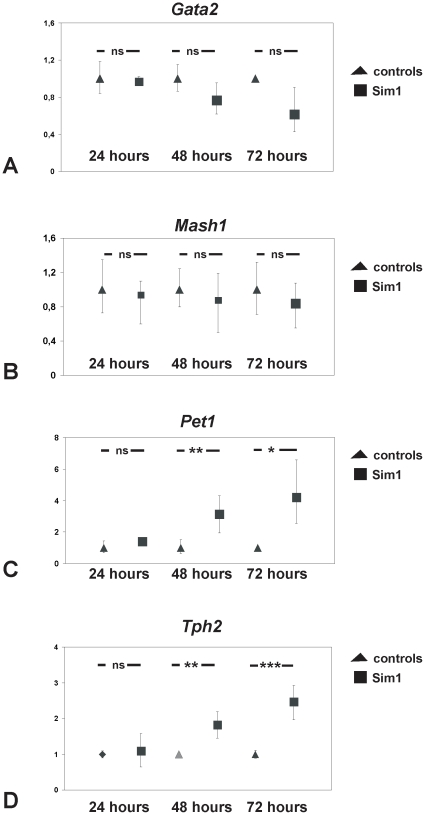
Differential regulation of serotonergic lineage determinants by *Sim1*. Quantitative real-time PCR analysis of *Gata2* (A), *Mash1* (B), *Pet1* (C) and *Tph2* (D) mRNA levels in pcDNA3::Sim1-transfected MN9D cells and in pcDNA3-transfected cells. Expression of individual gene is shown as 2^−ΔΔCt±s^. *Gata2* and *Mash1* expression remained unchanged in pcDNA3::Sim1-transfected MN9D cells, compared to pcDNA3-transfected cells 24 hours, 48 hours, and 72 hours after transfection. In contrast, *Pet1* and *Tph2* expression was significantly up-regulated in pcDNA3::Sim1-transfected MN9D cells, compared to the controls 48 hours and 72 hours after transfection (*p<0.05; **p<0.01; and ***p<0.0001 using the Student's *t*-test, n = 3).

To further understand the mechanism underlying the effect of *Sim1* on serotonergic neurons, we investigated whether expression of early and late determinants of the serotonergic lineage might be influenced by *Sim1*. Fig. 5 illustrates quantitative real-time PCR (qRT-PCR) analysis of relative expression of *Gata2*, *Mash1*, *Pet1,* and *Tph2* in MN9D cells transfected either with control or *Sim1* expression vectors 24 h, 48 h, and 72 h. *Sim1* overexpression had no effect on *Gata2* (A) and *Mash1* (B) expression at all time points. By contrast, as shown in Fig. 5C, expression of *Pet1*, a transcription factor exclusively expressed in postmitotic 5-HT neurons, was found significantly upregulated in Sim1 overexpressing MN9D cells, relative to the controls, at 48 h and 72 h after transfection (**p = 0.003 and *p = 0.01 for 48 h and 72 h, respectively; n = 3). Moreover, expression of *Tph2* (D), the rate-limiting enzyme in brain serotonin biosynthesis, was significantly upregulated in Sim1 overexpressing cells, compared to the controls, at 48 h and 72 h as well (**p = 0.006 and ***p = 0.0009 for 48 h and 72 h, respectively; n = 3). These data indicate that *Pet1* and *Tph2* expression is likely controlled by Sim1, either directly or indirectly.

### Expression of *Sim1* target genes *RGS4*, *Lhx8*, and *Brn3.2*, is upregulated in ventral hindbrain

**Figure 6:**
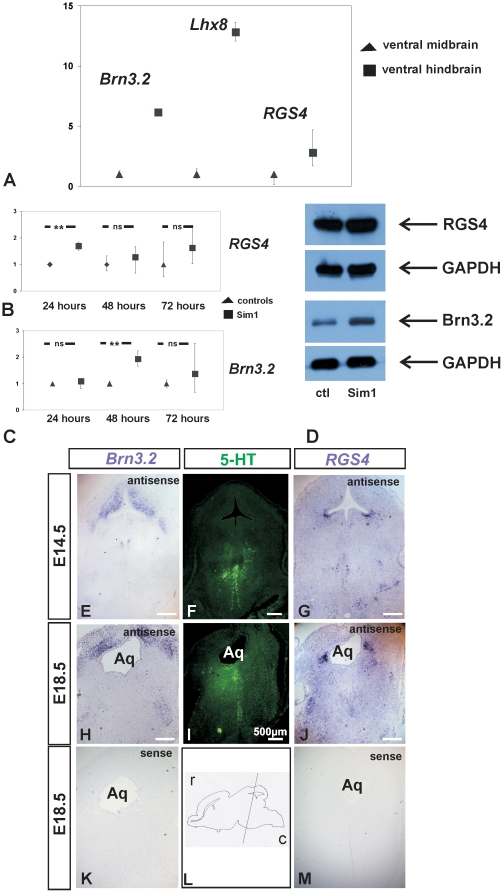
Identification of candidate genes involved in the development of serotonergic neurons. A: Quantitative real-time PCR analysis showed that expression of the transcription factors *Brn3.2* and *Lhx8*, and of a regulator of G protein signalling and modulator of 5-HT_1A_-mdiated neurotransmitter release, *RGS4,* are significantly upregulated in mouse ventral hindbrain tissue compared to ventral midbrain primary tissue at embryonic day 11.5. B-C: Regulation of expression of candidate genes by Sim1, assessed by quantitative real-time PCR. *Rgs4* (B) and *Brn3.2* (C) mRNA levels in pcDNA3::Sim1-transfected MN9D cells and in pcDNA3-transfected cells were analyzed. Expression of individual gene is shown as 2^−ΔΔCt±s^. *Rgs4* and *Brn3.2* expression was significantly up-regulated 24 hours and 48 hours, respectively, after transfection of MN9D cells with pcDNA3::Sim1 expression vector, compared with the controls (**P<0.01, using the Student's *t*-test, n = 3). D: Immunoblotting for RGS4 and Brn3.2 protein abundance in homogenates of MN9D cells transfected either with pcDNA3::Sim1 or pcDNA3 expression vectors (ctl) 48 hours after transfection. The immunoblots were probed either with monoclonal antibody against GAPDH or with a goat polyclonal antibody against RGS4 or Brn3.2. RGS4 protein expression was comparable between controls and pcDNA3::Sim1-transfected MN9D cells. In contrast, Brn3.2 protein abundance was significantly upregulated in pcDNA3::Sim1-transfected MN9D cells, compared to the pcDNA3-transfected cells. (**p<0.01 after densitometric analysis of the signal ratio Brn3.2: GAPDH and Student's *t*-test; n = 3). The blots are representative for three different experiments. 30 µg protein was loaded per lane. E, G: In situ hybridization for expression of candidate genes in mouse at E14.5 using antisense probes. *Brn3.2* expression was present in hindbrain, but not in the area of rostral serotonergic neurons (E), whereas *RGS4* expression was detectable in rostral serotonergic neurons (G). F: immunofluorescence for 5-HT at E14.5. H, J: In situ hybridization for expression of candidate genes in mouse at E18.5 using antisense probes. *Brn3.2* expression was present in hindbrain, but not in the area of rostral serotonergic neurons (H), whereas *RGS4* expression was detectable in rostral serotonergic neurons (J). I: immunofluorescence for 5-HT at E18.5. K, M: In situ hybridization at E18.5 for expression of *Brn3.2* (K) and *RGS4* (M) using sense probes revealed no detectable staining. L: schematic presentation of a sagittal section of mouse brain at E18.5 with a line drawn to indicate the approximate level of brain sections used for H-M (r: rostral, c: caudal). Aq: aqueduct.

To investigate the difference between the transcriptional profiles between rostral 5-HT and mDA at their early stages of development we have screened for genes that are upregulated in mouse ventral hindbrain, compared to ventral midbrain tissue at E11.5 and therefore could be considered as potential candidates involved in the induction of 5-HT neurons. Fig. 6A shows qRT-PCR analysis and relative mRNA expression of the transcription factors *Brn3.2* and *Lhx8*, as well as for *RGS4*, a regulator of G protein signalling and modulator of 5-HT_1A_-mediated neurotransmitter release, in mouse E11.5 ventral hindbrain tissue, compared to ventral midbrain. The results showed significant upregulation of *Lhx8* by approximately 12.82 fold in mouse ventral hindbrain, compared to ventral midbrain. Expression of *Brn3.2* was found ∼6.16 fold upregulated in ventral hindbrain. Finally, *RGS4* expression was upregulated by approximately 2.81 fold in the ventral hindbrain, thus considerably lower than *Lhx8* and *Brn3.2*, but still significantly higher than in mouse ventral midbrain.

Since *RGS4* and *Brn-2,* a homolog of *Brn3.2*, have been identified as target genes of *Sim1* [13], [27], their expression has been investigated in MN9D cells transfected with pcDNA3::Sim1 expression vector and compared with the controls by qRT-PCR. As illustrated in Fig. 6B, *Sim1* overexpression in MN9D cells resulted in a significant increase of *RGS4* expression 24 h after transfection (**p = 0.003; n = 3). This upregulation was however transient, since 48 h and 72 h after transfection *RGS4* expression was found comparable to the controls. Fig. 6C represents qRT-PCR analysis for *Brn3.2*, in controls and *Sim1* overexpressing cells. *Brn3.2* mRNA expression level showed no significant differences between the experimental groups 24 h after transfection. However, 48 h after transfection, *Brn3.2* was significantly upregulated in *Sim1*-overexpressing cells, compared with the MN9D cells transfected with pcDNA3 vector (**p = 0.0023; n = 3). Regulation of RGS4 and Brn3.2 by Sim1 has been further investigated at the protein level by immunoblotting (Fig. 6D) in homogenate of MN9D cells transfected with pcDNA3::Sim1 expression vector and compared to the controls, 48 h after transfection. The results show comparable protein levels of RGS4 between controls and Sim1 overexpressing cells, confirming the qRT-PCR data. In contrast, Brn3.2 protein was found upregulated 48 h after transfecting MN9D cells with pcDNA3::Sim1 expression vector, compared to the controls.

To assess whether the upregulated genes *Brn3.2* and *RGS4* were expressed in rostral serotonergic neurons, in situ hybridization has been performed on mouse fixed cryosections. Figs. 6E-6G illustrate coronal sections from E14.5 mouse embryonic brain and Figs. 6H-6J, 6K, and 6M illustrate coronal sections from E18.5 mouse embryonic brain at the level of pontine dorsal raphe (Fig. 6L). Using a *Brn3.2* antisense probe, at both E14.5 (E) and E18.5 (H) *Brn3.2* was predominantly expressed in areas of hindbrain lacking serotonergic neurons, thus showing no co-localization with 5-HT, illustrated by immunofluorescence in the respective consecutive sections (Figs. 6F and 6I). Labeling was considered specific, since incubation of the sections with sense probe (K) revealed no detectable *Brn3.2* labeling. In contrast, distinct 5-HT immunoreactive neurons were additionally positive for *RGS4*, at E14.5 as well as at E18.5, as shown in Figs. 6G and 6J using an antisense RGS4 in situ hybridization probe. However, *RGS4* expression pattern was not restricted to 5-HT neurons. Other cell types were positive for *RGS4* as well. When sections were incubated with *RGS4* sense probe, no labeling could be detected (Fig. 6M).

### RGS4, Brn3.2, and Pet1 are target genes of Sim1 in vivo

**Figure 7:**
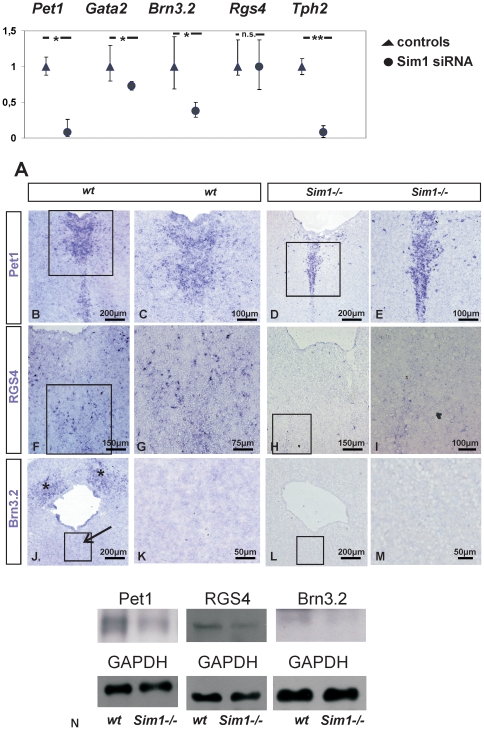
*Sim1* loss-of-function *in vitro* and *in vivo*. A: Quantitative real-time PCR analysis for candidate genes in MN9D cells transfected either with control siRNA (ctl) or with specific *Sim1* siRNA. Gene expression is shown as 2^−ΔΔCt±s^. *Pet1, Gata2, Brn3.2,* and *Tph2*, but not *RGS4* expression was significantly down-regulated in *Sim1* siRNA-transfected cells, compared to the controls, 24 hours after transfection (*p<0.05 and **p<0.01, using the Student's *t*-test, n = 3). B-M: In situ hybridization for *Pet1* (B-E), *RGS4* (F-I) and *Brn3.2* (J-M) in wild type (*wt*) and *Sim1-/-* mutants at P0. *Pet1* expression was observed in the area of rostral 5-HT neurons (B) including the area of dorsal raphe in *wt* (C: magnification of the inset in B), but was considerably decreased in *Sim1-/-* (D). E: magnification of the inset illustrating the area of the dorsal raphe. *RGS4* (F, and magnification of the inset in G) was also expressed in the dorsal raphe in *wt*, and was considerably decreased in *Sim1* mutants (H and magnification of the inset in I). In contrast, although *Brn3.2* expression in *wt* was present (J, asterisks) the area of rostral 5-HT neurons was devoid of *Brn3.2* expression (J, arrow and magnification of the inset in K). In *Sim1-/-* mutants *Brn3.2* expression was absent (L, and magnification of the inset in M). N: immunoblot analysis for Pet1, RGS4, and Brn3.2 in hindbrain of *wt* and *Sim1-/-* mutants. Pet1, RGS4, and Brn3.2 protein abundance was significantly downregulated in *Sim1-/-* hindbrain, compared to *wt.* (*p<0.05 after densitometric analysis of the signal ratio Pet1:GAPDH, RGS4:GAPDH or Brn3.2:GAPDH and Student's *t*-test; n = 4). The blots are representative for four different experiments. 30 µg protein was loaded per lane.

The results obtained from the *Sim1* gain-of-function experiments were further verified by *Sim1* loss-of-function experiments. Therefore, MN9D cells were transiently transfected with *Sim1* specific siRNA, coupled to CY5. 24 h after transfection and subsequent FACS, transfected cells were processed for total RNA isolation, cDNA synthesis and subsequent qRT-PCR using *Pet1, Gata2, Brn3.2, Rgs4,* and *Tph2* specific primers. As shown in Fig. 7A, *Pet1, Gata2, Brn3.2, and Tph2* expression was significantly downregulated 24 h after transfection of MN9D cells with *Sim1* specific siRNA, compared to MN9D cells transfected with control siRNA. In contrast, *Rgs4* expression 24 h after downregulation of Sim1 remained comparable with that of the controls. Together with the gain-of-function results, these data suggest *Pet1, Brn3.2* and *Tph2* as downstream targets of *Sim1* (*P<0.05 for *Pet1, Gata2, and Brn3.2*, and **P<0.01 for *Tph2*, n = 3).

The biological significance of the *in vitro* data has been further validated *in vivo.* Therefore, we have used fixed brain tissue sections from *wt* and *Sim1-/-* at P0 and performed in situ hybridization for *Pet1* (Figs. 7B-7E), *RGS4* (Figs. 7F-7I) and *Brn3.2* (Figs. 7J-7M). In sections from *wt* mice at P0, *Pet1* mRNA expression appeared distinct and of moderate intensity (Fig. 7B). At higher magnification (Fig. 7C) the area of the DRN is clearly positive for *Pet1*. In sections from *Sim1-/-* brains at P0, labeling for *Pet1* was decreased in DRN, as shown in Figs. 7D and 7E, suggesting *Pet1* as downstream target of *Sim1*. Similarly, as shown in Figs. 7F-7I, *RGS4* expression was detected in the area of rostral serotonergic neurons in *wt*, whereas in *Sim1-/-* mutants (Figs. 7H and 7I) *RGS4* labeling was absent, demonstrating downregulation *in vivo*. *Brn3.2* mRNA expression is shown in Figs. 7J-7M. Although *Brn3.2* labeling of strong intensity could be observed in *wt* (Fig. 7J; asterisks), the area of rostral serotonergic neurons, including dorsal raphe nucleus (Figs. 7J arrow, and 7K), and other raphe nuclei, were devoid of *Brn3.2* expression. In *Sim1-/-* (Figs. 7L and 7M) *Brn3.2* expression was completely abolished, demonstrating that *Brn3.2* is a target gene of *Sim1* as well.

Finally, to validate the mRNA data at the protein level, protein extraction has been performed from 4%-fixed cryosections from hindbrain of *wt* and *Sim1-/-* mutants, as described in Material and Methods, followed by western blot analysis. As illustrated in Fig. 7N, Pet1, RGS4 and Brn3.2 protein abundance was significantly downregulated in *Sim1-/-* compared to the *wt* (0.57±0.25 fold, *p = 0.032, 0.67±0.12, *p = 0.048 and 0.70±0.05, *p = 0.044, for Pet1, Rgs4 and Brn3.2, respectively, n = 4,)

## Discussion

In the present study we have addressed the question whether the mouse homologue of the Drosophila *sim*, mSim1, might be involved in the differentiation of two clinically important ventral neuronal populations, namely the mDA and rostral 5-HT neurons. These neuronal subpopulations reveal positional and developmental relationships and share common transcriptional determinants during their differentiation and specification.

After having shown that Sim1 is expressed in TH positive and 5-HT immunoreactive cells, we further investigated the biological significance of Sim1 during the development of mDA and 5-HT neurons *in vivo*, by examining *Sim1* deficient mice at E14.5 and P0. Since *Sim1* mutants die shortly after birth due hypothalamic developmental defects [27], [28], an investigation in adult animals was not possible. Our results demonstrate comparable number of mDA in *Sim1* mutants and wild type at both stages examined. In contrast, Sim1 has been shown to be involved in the differentiation of zebrafish diencephalic A11-related dopaminergic neurons [29]. Gene ablation of *Sim1* had no effect on the total number of rostral 5-HT neurons at E14.5 as well, a time point in which individual rostral nuclei raphe cannot be distinguished. However, in *Sim1-/-* newborn mice the number of 5-HT neurons in the dorsal raphe nucleus (DRN) was significantly decreased by 30% (Fig. 2J), but not in the median and paramedian raphe, demonstrating a selective effect of Sim1 on a rostral serotonergic subpopulation. The serotonergic system is involved in the modulation and coordination of a variety of autonomic functions and behaviour, including the sleep-wake cycle, aggression, anxiety, mood, and stress. Besides, or even due to, its different functions the serotonergic system is characterized by extensive and complex cellular heterogeneity. First, 5-HT neurons consist of nine cell groups (B1-B9) that are separated into two clusters, the rostral (B5-B9) and caudal (B1-B4) nuclei raphe. Second, rostral and caudal 5-HT neurons are differentially regulated by developmentally potent signaling molecules, such as TGF-β [30], and also exhibit different electrophysiological properties, suggesting that they likely represent developmentally and functionally distinct neuronal subpopulations. Moreover, even within a given raphe nucleus individual cell subpopulations exist, characterized by different developmental background and topographically organized efferent projections. In particular, the neurons of DRN vary considerably with regard to their synaptic morphology and their electrophysiological properties from each other [31], [32]. The DRN of the brainstem is the main source of serotonergic innervation of limbic structures fundamental in the regulation of emotionally influenced behaviour. Interestingly, serotonergic neurons that innervate the central nucleus of the amygdala, and subsequently exert a modulatory action on the vegetative centers of the hypothalamus, are restricted to the middle and caudal portions of the DRN [33]. Whether the observed loss of DRN 5-HT neurons in *Sim1* mutants is restricted to a distinct neuronal subpopulation within this nucleus is not clear. Nevertheless, our results provide evidence that differential developmental transcriptional networks likely correspond to each neuronal subpopulation. Elucidation of the putative mechanism underlying Sim1 effects on rostral 5-HT neurons has been performed by gain- and loss-of-function experiments using the MN9D cells line as a culture system. The gene expression analysis performed in the present study (Fig. 3A) clearly demonstrates that these cells express determinants of the serotonergic lineage, represented by *Gata2*, *Pet1,* and *TPH2*. The transcription factor *Pet1* together with *Lmx1b, Nkx2.2, Mash1, Gata2, Gata3,* and *Phox2b* form a transcriptional network, which specifies the differentiation of serotonergic neurons from hindbrain progenitors at E11 in the mouse. According to the current view for serotonergic neurons differentiation, *Phox2b* is switched off at E10.5. Together with *Mash1* and *Nkx2.2,* the transcription factors *Gata3, Gata2, Lmx1b, and Pet1* are activated, which subsequently define the cell fate by activating serotonergic lineage marker genes, such as *TPH2*. However, phenotype analyses from gene ablations studies have demonstrated that the suggested transcriptional network is still not complete and additional determinants are involved in the specification of rostral 5-HT neurons. Based on the results of the *in vitro* experiments, several lines of evidence propose *Sim1* as a putative novel determinant in the transcriptional network orchestrating 5-HT neuron development. Sim1 overexpression did not affect expression of *Gata2* though (Fig. 5A), a mitotic and postmitotic marker for rostral serotonergic neurons. This observation is quite surprising, since in a previous study in Neuro2A cells *Gata2* has been identified as a downstream target of Sim1 [22]. Along this line, transfection of MN9D cells with specific *Sim1* siRNA significantly downregulated *Gata2* expression (Fig. 7A), demonstrating a role of Sim1 in maintenance of *Gata2* expression. *Sim1* overexpression had no effect on *Mash1* as well, an early determinant of the transcriptional network underlying 5-HT development [9]. By contrast, *Sim1* was able to activate *Pet1* and *Tph2*, late markers specific for the serotonergic lineage. Both *Pet1* and *Tph2* were significantly upregulated after Sim1 overexpression, and significantly downregulated after transfection of MN9D cells with specific Sim1 siRNA, implicating *Pet1 and Tph2* as putative downstream targets of *Sim1*. Moreover, conserved Pet1 binding sites are present in the promoter of several genes characteristic for the serotonergic phenotype, among them TPH2 [11], thus suggesting a putative alternative indirect cascade for *Tph2* activation, by means that *Tph2* regulation by *Sim1* occurs secondarily through *Pet1.* In addition, in *Sim1-/-* only a subpopulation of 5-HT neurons was affected, and in *Pet1-/-* mutants 5-HT neurons were dramatically decreased but not completely lost, strongly suggesting that more than one pathway should be involved in the differentiation of 5-HT neurons. It is conceivable that *Pet1* can be regulated by multiple transcription inputs at different subpopulation of 5-HT neurons. Along this line, we have revealed that for the dorsal raphe nucleus *Sim1* is a crucial regulator. Notably, it has recently been shown that Pet1-dependent and Pet-1-resistant 5-HT neurons exist, that have different morphological features, including targeting of different brain areas and formation of synaptic junctions [34].

We have also demonstrated that *RGS4*, *Lhx8,* and *Brn3.2*, all downstream targets of *Sim1*, are significantly upregulated in mouse E11.5 ventral hindbrain, compared to ventral midbrain (Fig. 6A). *Sim1* overexpression in MN9D cells significantly upregulated *RGS4* (Fig. 6B). Consistently, *RGS4* expression (Figs. 7H, 7I) and protein abundance (Fig. 7N) was downregulated in hindbrain of *Sim1-/-* mutants. This result confirms and extends previous observations, in which expression of *RGS4* is downregulated in hypothalamic domains in *Sim1-/-* mice [27]. Although the *in vivo* roles of RGS4 are not fully understood, it has been identified to have the capacity to modulate neurotransmitter receptors in the rat brain. Interestingly, RGS4 modulates 5-HT_1A_-mediated neurotransmitter release, *in vitro* and *in vivo* [35], [36]. In the context of the present work, this observation is of particular interest, since 5-HT_1A_ represent the autoreceptors in the DRN. Taken into consideration that 5-HT can also affect differentiation, RGS4 expression during early 5-HT neuron development could be critical for terminal differentiation of dorsal raphe 5-HT neurons. Indeed, as shown in Figs. 6G and 6J, a colocalization of 5-HT and RGS4 *in vivo* was apparent in mouse hindbrain.

In addition, after *Sim1* overexpression, expression of a class IV POU domain transcription factors member, *Brn3.2* (POU4F2), was found upregulated as well (Fig. 6C, 6D). Members of the class IV POU domain proteins are required for normal differentiation and survival of several neuronal populations [37]. The present study provides first evidence that *Brn3.2* is downregulated in *Sim1-/-* mutants (Figs. 7L, 7M, 7N). However, *Brn3.2* expression was restricted to areas of the hindbrain outside the serotonergic system, suggesting that differentiation of other neuronal subpopulations of the hindbrain, but not 5-HT neurons, might be controlled by *Brn3.2*. In contrast, *Brn-2* (also known as POU3F2) has been identified to act downstream of *Sim1* during anterior hypothalamus development [20], and has been shown to be expressed in rat adult DRN, where it colocalizes with TPH2. Importantly, *Brn-2* activates the *Tph2* promoter [38]. These observations, together with results of the present study, where Sim1 overexpression upregulated *Tph2* expression (Fig. 5D), suggest a putative interaction of *Sim1* with *Tph2* via *Brn-2* and provide an attractive scenario for putative mechanisms underlying the phenotype observed in *Sim1 -/-* newborn mice.

Reduced expression of serotonergic markers such as TPH2 resembles observations obtained in the dopaminergic system of zebrafish, where in the absence of Arnt2/Sim1 function, the dopaminergic markers *th*, *dat* and *ddc* were reduced in posterior tuberculum and hypothalamus [29]. Taken into consideration that Sim1 has been described as a transcriptional repressor, further studies are needed to clarify the molecular interactions and pathways leading to activation of transcription factors involved in the terminal specification of serotonergic neurons. As an example, another possible scenario could imply that Sim1 may act indirectly by repressing inhibitors of serotonergic lineage.

In conclusion, the results of the present study strongly suggest Sim1 involvement in terminal differentiation of rostral 5-HT phenotype in at least two different processes. First, Sim1 acts on the development of the DRN through a developmental pathway leading to *Tph2* activation via *Brn-2*, additionally or alternatively via *Pet1*. Second, Sim1 acts to modulate serotonin release of the B7 group of 5-HT neurons via regulating *RGS4*. As *RGS4* is only transiently up-regulated by *Sim1*, there likely exists a feedback loop to maintain a proper level of RGS4 and hence the homeostasis of serotonin release. Our study underscores that subpopulation of a common neurotransmitter phenotype use distinct combinations of transcription factors to control the expression of shared properties i.e. Pet1 and Tph2.

## Materials and Methods

This study was carried out in strict accordance with the National Health and ethical regulations and care of animals was in accordance with institutional guidelines. The protocol was approved by the Committee on the Ethics of Animal Experiments of the University of Freiburg (Permit Number: X-07/27A). Animals were sacrificed under sodium pentobarbital anesthesia and all efforts were made to minimize suffering.

### RT-PCR

Total RNA was isolated from tissue of mouse ventral midbrain, ventral hindbrain, and isthmus, reverse transcribed and processed for PCR [39]. The forward and reverse primer sequences used are listed in Table 1.

**Table 1:** Primer sequences used for RT-PCR gene expression analysis.

Primer	Accession	PCR-fragment
*Gapdh* F: 5′-CGGCCGCATCTTCTTGTG-3′	NM008084	nt 804-1210
*Gapdh* R: 5′-TGACCAGGCGCCCAATAC-3		
*Nestin* F: 5′-CAGGCTTCTCTTGGCTTTCCTG-3′	NM016701	nt 1074-1512
*Nestin* R: 5′-GGTGAGGGTTGAGGGGTGG-3′		
*Gata* 2 F: 5′-CACCCCGCCGTATTGAATG-3′	NM008090	nt 37-147
*Gata 2* R: 5′-CCTGCGAGTCGAGATGGTTG-3′		
*Mash1* F: 5′-ACTTGAACTCTATGGCGGGTT-3′	NM008553	nt 584-690
*Mash1* R: 5′-CCAGTTGGTAAAGTCCAGCAG-3′		
*Pet1* F: 5′- AGCAAGCCTAACATGAACTACG	NM153111	nt 484-593
*Pet1* R: 5′-AAGTCAAAGCGGTAGGCG-3′		
*Sim1* F: 5′-CTTTCTTTTATACTTACACCTCACGTTTTC-3′	NM011376	nt 37-152
*Sim1* R: 5′-AACATAACTTTAAACAGGAGGCTGAAG-3′		
*Sim1* F: 5′-TGAAGTGTGTTTTGGCCAAGC-3′	NM 011376	nt 736-1052
Sim1 R: 5′-ATGCACGTGATGGTACAGGGT-3′		
*Tph2* F: 5′-AAGTCGAAATCTTCGTGGACTG-3′	NM173391	nt 432-531
*Tph2* R: 5′-GGCGGATTCAGGGTCACAAT-3′		
*Brn3.2* F: 5′-ACATCGTCTCCCAGAGTAAGAG-3′	NM138944.2	nt 607-703
*Brn3.2* R: 5′-CACGGGATGGTGTTCTGG-3′		
*Lhx8* F: 5′-ACACGAGCTGCTACATTAAGGA-3′	NM010713.2	nt 606-716
*Lhx8* R: 5′-CCCAGTCAGTCGAGTGGATG-3′		
*RGS4* F: 5′-GAGTGCAAAGGACATGAAACATC-3′	NM009062.2	nt 139-291
*RGS4* R: 5′-TTTTCCAACGATTCAGCCCAT-3′		

(nt: nucleotide).

For detection of cDNAs encoding the respective proteins, following protocol was used: denaturation at 95°C for 5 minutes, the optimum number of cycles -depending on primer pair- of PCR amplification were performed at following conditions: denaturation at 94°C for 1 minute, annealing at appropriate temperature for 1 minute, and elongation at 72°C for 1 minute. Final extension at 72°C for 10 minutes was terminated by rapid cooling at 4°C.

### Cell culture

The MN9D cell line, a hybridoma cell line established by fusing embryonic primary cells from mouse ventral midbrain with cells from the mouse neuroblastoma cell line N18TG2 [25] was used for *in vitro* experiments. Cells were cultured in DMEM/F-12 1∶1, supplemented with 5% FCS, and 50 µg/ml gentamycin, passaged when confluent, and incubated in a 95% air /5% CO_2_ atmosphere at 37°C. One day before transfection cells were plated at a density of 1×10^6^ cells in 25 cm^2^ flasks. Cells were transfected with expression vectors pcDNA3 or pcDNA3::Sim1, 6 µg of plasmid DNA per flask. Transfection was performed with Lipofectamine (Invitrogen, Karlsruhe, Germany) according to the manufacturer's protocol. After 4 h of incubation with DNA precipitate, complete medium containing 5% FCS was added. 24 h, 48 h, and 72 h post transfection cells were harvested and probes were processed for either RNA isolation and subsequent qRT-PCR or immunoblotting.

Alternatively, cells were seeded at 1×10^6^ cells in 25 cm^2^ flasks and transiently transfected with CY5-labeled siRNA targeting mouse *Sim1* mRNA (purchased from Qiagen). The siRNA was transiently transfected into MN9D cells with 750 ng siRNA in 30 µl HiPerFect reagent (Qiagen). A sequence that reveals no homology with any known mammalian genes has been labeled with Alexa 488 and was used as control siRNA. Cells were harvested and lysed 24 h after transfection, and gene expression was analyzed by qRT-PCR.

### FACS analysis of transfected MN9D cells with *Sim1* siRNA

MN9D cells successfully transfected either with CY5-labeled siRNA targeting mouse *Sim1* or with control siRNA labeled with Alexa 488 were analyzed by flow cytometry. Briefly, MN9D cells were sedimented (820 *g*, 10 minutes, 4°C), resuspended with DMEM-F12, containing 2% FCS and 10 mM HEPES. Cells were incubated at 37°C for 90 minutes, centrifuged, and resuspended with HBSS containing 2% FCS and 10 mM HEPES. Cells were then processed for RNA isolation and subsequent qRT-PCR.

### Real-Time PCR

Total RNA was isolated from MN9D cells transiently transfected with either pcDNA3 or pcDNA3::Sim1 expression vectors and subsequently 1.0 µg was reverse-transcribed. The primers used for qRT-PCR are listed in Table 1.

Real-time PCR was performed as described elsewhere [40]. Cycle conditions: denaturation at 95°C for 10 minutes, and 40 cycles of PCR amplification at 95°C for 30 seconds and 56°C for 30 seconds and elongation at 72°C for 1 minute. All PCRs were performed in triplicate on an ABI PRISM 7500 Sequence Detection System.

The mean ± SD of the Ct values for the genes of interest and GAPDH were determined and statistically analyzed using the Student's *t*-test. *p<0.05 was considered as statistically significant. For documentation of the data, relative mRNA levels were calculated using the comparative C_t_ method (2^−ΔΔCt^), as previously reported [39].

### Immunoblotting

MN9D cells were harvested and homogenized, and protein concentration was determined according to Bradford [41]. For isolating proteins from hindbrain of 4%-PFA fixed brain cryosections from wildtype and *Sim1-/-* mutants, the method described by Scicchitano et al., [42] has been applied, using the Qproteome FFPE Tissue Kit from Qiagen, following the manufacturer's instructions. After determination of protein concentration, electrophoresis and blotting procedures were performed as described [43]. Blots were incubated with primary antibody overnight at dilution 1∶1,000 for Sim1 (rabbit polyclonal; Chemicon; raised against amino-acids 423-435 of human Sim1), 1∶1,000 for TH (mouse monoclonal, Chemicon, Hofheim, Germany), 1∶500 for Pitx3 (rabbit polyclonal; Chemicon), 1∶1,000 for TPH2 (mouse monoclonal, Sigma, Taufkirchen, Germany;), 1∶1,000 for Pet1 (rabbit polyclonal, Abcam, Cambridge, UK), 1∶500 for RGS4 and Brn3.2 (goat polyclonal, from Santa Cruz), and 1∶10,000 for GAPDH (mouse monoclonal, Abcam). After incubation with secondary antibodies, blots were developed in enhanced chemiluminescence reagents and signals were visualized on X-ray film. Subsequently, films were scanned and the signal ratio TH:GAPDH, Pitx3:GAPDH, TPH2:GAPDH, Pet1:GAPDH, RGS4:GAPDH and Brn3.2:GAPDH was quantified densitometrically. Differences in signal ratio were tested for significance using Student's *t*-test. Results with levels of *p<0.05 were considered significant.

### Animals

The generation of *Sim1-/-* mice has been described previously [20]. For morphological comparison wild type littermates were used as wild type controls. The morning of the day on which a vaginal plug was detected in mating was designated gestation day 0.5. Analysis was performed on 4% paraformaldehyde-fixed 14.5-day-old embryos (E14.5) and in newborn mice (P0). For immunohistochemistry experiments embryos or heads of newborn mice were dehydrated, embedded in paraffin, and cut into 10 µm serial sections. For in situ hybridization, they were cryoprotected and cut into 10 µm cryosections.

### Immunohistochemistry

Immunohistochemistry has been performed as described earlier [43]. Monoclonal mouse anti-TH (1∶100; Chemicon), rabbit polyclonal anti-5HT antibody (1∶1,000; Sigma) or rabbit polyclonal anti-Sim1 (1∶400) were used as primary antibodies and goat anti-mouse or goat anti-rabbit IgG coupled to either horseradish peroxidase, or FITC as secondary antibodies.

Numbers of TH- and 5-HT-labeled neurons were counted on the complete series of 10 µm transverse sections after immunoperoxidase and immunofluorescence staining. A neuron was designated as TH or 5-HT positive if it revealed a darkly labeled cytoplasm and a clearly visible, unstained nucleus. Only cells fulfilling these criteria were included in the cell counts. To avoid double counting the same cell on two sequential sections, only every fifth section was counted.

### *In situ* hybridization

Non-radioactive in situ hybridization on cryosections and preparation of digoxigenin-labelled probes for *RGS4* [35], *Pet1* [11] and *Brn3.2* [44] were carried out as described by Ernsberger et al. [45].

### Statistics

Data are presented as the mean ± SD. Statistical analysis was performed using the Student's unpaired *t*-test. Differences were considered statistically significant at *p<0.05, **p<0.01 and ***p<0.001.
